# The effect of CFTR modulators on structural lung disease in cystic fibrosis

**DOI:** 10.3389/fphar.2023.1147348

**Published:** 2023-04-11

**Authors:** L. Clara Mok, Antonio Garcia-Uceda, Matthew N. Cooper, Mariette Kemner-Van De Corput, Marleen De Bruijne, Nathalie Feyaerts, Tim Rosenow, Kris De Boeck, Stephen Stick, Harm A. W. M. Tiddens

**Affiliations:** ^1^ Faculty of Medicine and Health Sciences, The University of Western Australia, Perth, WA, Australia; ^2^ Telethon Kids Institute, The University of Western Australia, Nedlands, WA, Australia; ^3^ Department of Radiology and Nuclear Medicine, Erasmus Medical Center, Rotterdam, Netherlands; ^4^ Department of Pediatric Pulmonology and Allergology, Erasmus Medical Center-Sophia Children’s Hospital, Rotterdam, Netherlands; ^5^ Department of Computer Science, University of Copenhagen, Copenhagen, Denmark; ^6^ Department of Pediatric Pulmonology, University of Leuven, Leuven, Belgium; ^7^ Department of Respiratory Medicine, Perth Children’s Hospital, Perth, WA, Australia

**Keywords:** CFTR modulator, chest CT, quantitative measures, structural lung disease, cystic fibrosis

## Abstract

**Background:** Newly developed quantitative chest computed tomography (CT) outcomes designed specifically to assess structural abnormalities related to cystic fibrosis (CF) lung disease are now available. CFTR modulators potentially can reduce some structural lung abnormalities. We aimed to investigate the effect of CFTR modulators on structural lung disease progression using different quantitative CT analysis methods specific for people with CF (PwCF).

**Methods:** PwCF with a gating mutation (Ivacaftor) or two Phe508del alleles (lumacaftor-ivacaftor) provided clinical data and underwent chest CT scans. Chest CTs were performed before and after initiation of CFTR modulator treatment. Structural lung abnormalities on CT were assessed using the Perth Rotterdam Annotated Grid Morphometric Analysis for CF (PRAGMA-CF), airway-artery dimensions (AA), and CF-CT methods. Lung disease progression (0–3 years) in exposed and matched unexposed subjects was compared using analysis of covariance. To investigate the effect of treatment in early lung disease, subgroup analyses were performed on data of children and adolescents aged <18 years.

**Results:** We included 16 modulator exposed PwCF and 25 unexposed PwCF. Median (range) age at the baseline visit was 12.55 (4.25–36.49) years and 8.34 (3.47–38.29) years, respectively. The change in PRAGMA-CF %Airway disease (-2.88 (−4.46, −1.30), *p* = 0.001) and %Bronchiectasis extent (-2.07 (−3.13, −1.02), *p* < 0.001) improved in exposed PwCF compared to unexposed. Subgroup analysis of paediatric data showed that only PRAGMA-CF %Bronchiectasis (-0.88 (−1.70, −0.07), *p* = 0.035) improved in exposed PwCF compared to unexposed.

**Conclusion:** In this preliminary real-life retrospective study CFTR modulators improve several quantitative CT outcomes. A follow-up study with a large cohort and standardization of CT scanning is needed to confirm our findings.

## 1 Introduction

Respiratory disease begins early in life and is the primary cause of morbidity and mortality in people with cystic fibrosis (PwCF) (Davis, 2006). Progressive structural lung disease, including: airway wall thickening, mucus plugging, bronchiectasis and low attenuation regions can be observed in PwCF throughout life (Stick et al., 2009; Mott et al., 2012; Sly et al., 2013; Kuo et al., 2017a; Tiddens et al., 2020). Therefore, therapeutic intervention is needed to reduce or prevent the development of these irreversible structural changes. Ivacaftor (KalydecoTM, Vertex Pharmaceuticals) and lumacaftor-ivacaftor (OrkambiTM, Vertex Pharmaceuticals) are novel gene-specific CF transmembrane conductance regulator (CFTR) modulators, which increase chloride transport in patients with CFTR-gating mutations (Ramsey et al., 2011) or two Phe508del alleles (Boyle et al., 2014), respectively. Correcting CFTR dysfunction has been shown to reduce sweat chloride concentration (Boyle et al., 2014; Rosenfeld et al., 2018), pulmonary exacerbations (Flume et al., 2018; McColley et al., 2019), inflammation and infection (Hisert et al., 2017; Hubert et al., 2018) in addition to improving lung function (Ramsey et al., 2011; Ratjen et al., 2017), mucociliary clearance (Donaldson et al., 2018) and body mass index (BMI) (De Boeck et al., 2014; Wainwright et al., 2015) in PwCF. However, there has been limited work investigating the effects of CFTR modulators on structural lung disease (Sheikh et al., 2015; Chassagnon et al., 2016; Brody et al., 2017; Ronan et al., 2018; Dournes et al., 2021). Standard clinical trial outcome measures (e.g., spirometry) used in most of these studies are only indirectly related to structural lung damage (Ranganathan et al., 2001; de Jong et al., 2006; Ramsey et al., 2016), whereas lung imaging can provide additional information regarding the type, distribution and reversibility of lung disease.

Chest computed tomography (CT) is the current gold standard and most sensitive imaging tool to assess structural lung abnormalities, and is universally available. However, it is only recently that studies began to focus on using CT outcomes to assess the efficacy of novel, disease-modifying treatments (Sheikh et al., 2015; Chassagnon et al., 2016; Brody et al., 2017; Ronan et al., 2018; Dournes et al., 2021). To quantify disease severity in most of these studies, semi-quantitative CT scoring systems (e.g., CF-CT, Bhalla score) were used to assess different components of CF lung disease, including: bronchiectasis, airway wall thickening, mucus plugging and low attenuation regions (Sheikh et al., 2015; Chassagnon et al., 2016; Brody et al., 2017; Ronan et al., 2018). A disadvantage of these systems is their use of coarse categories, which leads to an inability to quantify small structural changes seen in CF airways. Therefore, more sensitive validated quantitative CT outcomes are required to detect changes in lung structure over time, as they have the potential reduce the size of samples in clinical trials and provide more incremental assessments of treatment efficacy in PwCF (Rosenow et al., 2015; Kuo et al., 2017b). Hsia et al. (2023) compiled relevant quantitative CT metrics for the assessment of pulmonary diseases.

Newly developed quantitative chest CT outcomes designed specifically to assess CF lung disease are now available. These include the Perth Rotterdam Annotated Grid Morphometric Analysis for CF (PRAGMA-CF) (Rosenow et al., 2015) method and objective measurements of airway-artery (AA) dimensions (Perez-Rovira et al., 2016; Kuo et al., 2017c), as well as other recent artificial intelligence methods (Dournes et al., 2021). Both PRAGMA-CF and AA-method provide sensitive and reproducible assessments of structural lung disease in PwCF (Rosenow et al., 2015; Kuo et al., 2017a). How well these measures perform in monitoring the effect of therapies like CFTR modulators has not yet been determined. Therefore, the aim of this real life study was to investigate the effects of CFTR modulator therapy on structural lung disease, using different CT analysis methods specific for CF lung disease. We hypothesised that quantitative CT outcomes would be sensitive to detect the effect of CFTR modulators on structural abnormalities related to CF lung disease.

## 2 Materials and methods

### 2.1 Study population

To maximise study power, a combined dataset consisting of coded data from the Perth Children’s Hospital (Perth, Australia), University Hospital of Leuven (Leuven, Belgium) and the Erasmus Medical Center Sophia Children’s Hospital (Rotterdam, the Netherlands) was used. Children and adults with a confirmed CF diagnosis who could contribute two volumetric (helical) or sequential routine chest CT scans made as part of the annual evaluation were included in this retrospective study. Demographic data including age, gender, height, CFTR genotype, pancreatic status, BMI, spirometry measurements and CFTR modulator treatment duration were also collected. Due to the retrospective nature of the study, written informed consent was waived by the relevant institutional human research ethics committees: Perth (Child and Adolescent Health Service Human Research Ethics Committee of Western Australia), Rotterdam (Medisch Ethische Toetsings Commissie Erasmus MC) and Leuven (Ethische Commissie Onderzoek UZ/KU Leuven).

### 2.2 Exposed subjects

Inclusion criteria: PwCF with at least one CFTR gating mutation or two Phe508del alleles and who initiated CFTR modulator therapy between the baseline CT and follow-up CT. Exposed PwCF with gating mutations received 150 mg Ivacaftor PO q12h with fat-containing food. Phe508del homozygous patients received 400 mg lumacaftor/250 mg ivacaftor PO q12h with fat-containing food. All patients received treatment in addition to their prescribed CF treatments.

### 2.3 Unexposed subjects

The unexposed subjects consisted of age, gender and where possible CFTR genotype-matched PwCF receiving standard clinical care, with no prior exposure to CFTR modulator therapy. Unexposed subjects were selected per treatment subject at a ratio of 1:1.5 (exposed:unexposed). The baseline CT scan of unexposed subjects was performed within a year of the scan of the matched modulator treated subject.

### 2.4 CT scanning

Chest CT was performed under general anaesthesia using endotracheal intubation (8 out of 82) (Stick et al., 2009), spirometer guidance (Ramsey et al., 2016), voluntary breath holds (Otjen et al., 2018) or free breathing as previously described. All scans were performed between 2007 and 2017.

### 2.5 CT image analysis

Chest CT scans were anonymised at each participating research centre prior to being sent and analysed at the LungAnalysis Core Laboratory, Erasmus Medical Center-Sophia Children’s Hospital (Rotterdam, the Netherlands). Only the inspiratory CT scan or functional residual capacity (FRC) scan for free breathing children were used in this study, as an expiratory scan was not routinely obtained for most subjects. Structural lung disease extent was assessed using three independent CT analysis methods, namely,: the PRAGMA-CF method (Rosenow et al., 2015), airway-artery dimensions (AA-method) (Perez-Rovira et al., 2016; Kuo et al., 2017c) and the CF-CT score (Brody et al., 2006). As the AA-method is sensitive to lung volume level, free breathing scans were not included in this analysis. Volumetric scans were required for analysis using the PRAGMA-CF and AA-method. Exclusion criteria for image analysis were as follows: inadequate image quality due to significant motion artefacts, or the absence of a reconstruction series needed for image analysis. Image analyses were performed by CF-CT and PRAGMA-CF certified experienced observers (NB and JB), and automated extraction of AA-dimensions and tapering measurements (AG-U). Observers NB and JB were trained using the standardised CF-CT training module developed by the LungAnalysis Core Laboratory, and PRAGMA-CF training module developed by the Australian Respiratory Early Surveillance Team for Cystic Fibrosis (AREST CF) and LungAnalysis. Image analysis of coded deidentified CTs by observers was executed in random order.

#### 2.5.1 PRAGMA-CF method

PRAGMA-CF is a quantitative CT analysis method that was initially developed for early CF lung disease (Rosenow et al., 2015), but has also been validated in more advanced lung disease (Bouma et al., 2020). Briefly, a grid is overlaid on 10 equidistant axial slices between lung apex and base. Grid cells are annotated in a hierarchical manner for the presence of 1. Bronchiectasis (%BE), 2. Mucus plugging (%MP), 3. airway wall thickening (%AWT), 4. atelectasis and 5. Normal lung on inspiratory scans. The lung volume proportion of each structural component was calculated, with atelectasis excluded from the total lung volume. Airway disease (%Dis) was calculated as the sum of %BE, %MP and %AWT. We reported the main outcomes (%Dis and %BE) and %MP, being related to inflammation and bronchiectasis (Tepper et al., 2016; Esther et al., 2019).

#### 2.5.2 AA-method and tapering

The AA-method has been developed to assess airway and artery dimensions in chest CT (Perez-Rovira et al., 2016; Kuo et al., 2017a). This method utilises the artery properties as a normalising factor for comparison of airway dimensions. Airways with an outer AA ratio (AAR) > 1.1 are considered to be bronchiectatic. The airway wall thickness-artery ratio (WAR) is used to assess bronchial wall thickening. To detect the lack of normal airway tapering, inner and outer wall intra- and inter-branch tapering are automatically extracted for each individual airway branch (Kuo et al., 2020). Intra-branch tapering (intra-BT) is defined as the progressive reduction in airway diameter along the branch. Inter-branch tapering (inter-BT) is defined as the reduction in airway diameter of a branch relative to the branch before bifurcation. Tapering measurements are presented as a percentage reduction of the airway diameter. Hence a reduced airway tapering can be used as an objective measure of bronchiectasis (Kuo et al., 2020).

In preparation for the automated analysis of AA-dimensions and tapering, approximated centrelines of the bronchial tree were manually traced by trained observers using specialist image analysis software (Myrian version 2.1.2, Intrasense, Montpelier, France). Next, 3D segmentations of the bronchial tree were automatically reconstructed around these centrelines for each CT scan using a surface graph-cut method as previously described (Petersen et al., 2014; Perez-Rovira et al., 2016). The segmentation of the vascular tree was segmented using a multi-scale Hessian Eigen analysis approach that detects elongated tubular structures (Petersen et al., 2014; Perez-Rovira et al., 2016). Airways and arteries were paired based on their similarity in orientation, proximity and size (Perez-Rovira et al., 2016). AA-pairs of airways that had an obstructed inner lumen (e.g., mucus plugging) and airways without a detectable paired artery (e.g., extensive atelectasis or severe bronchiectasis) were excluded from analysis, as they would provide unreliable measurements. Bronchoarteriolar dimensions (outer AAR, WAR) were automatically computed for each AA-pair. Intra- and inter-branch tapering measurements were computed using the inner and outer airway diameters (inner intra-BT, outer intra-BT, inner inter-BT, outer inter-BT) as previously described (Kuo et al., 2020). For ease of comparison with AA-dimensions, we only included tapering measurements of airways with a paired artery. As CF lung disease primarily affects the small airways (Tiddens et al., 2010; Kuo et al., 2017a; Kuo et al., 2017b), we reported median AA-dimensions and tapering measurements of airways with an accompanying artery diameter <3.08 mm (Kuo et al., 2020). We used normalized artery dimensions in order to compensate for differences in lung volume sizes in our cohort. Arteries are rescaled with a factor that accounts for the ratio between the actual patient’s lung volume and the average lung volume in the cohort, of approximately 4L.

#### 2.5.3 CF-CT scoring

The semi-quantitative CF-CT method is the most comprehensively examined CF-specific scoring system which evaluates the five lung lobes and lingula for structural lung abnormalities (Brody et al., 2006). Components assessed include: 1. extent and severity of bronchiectasis (central and peripheral), 2. bronchial wall thickening, 3. mucus plugging, 4. atelectasis/consolidation, and 5. bullae and cysts on inspiratory scans. The maximum achievable total score was 207. Sub-Scores were expressed as a percentage of the maximum achievable scores and ranged between 0 (no disease) and 100 (maximum lung disease). We reported bronchiectasis (%BE), mucus plugging (%MP) and airway disease (%Dis), as the sum of bronchiectasis, mucus plugging and bronchial wall thickening, to allow easy comparison with similar PRAGMA-CF outcomes.

### 2.6 Pulmonary function measures

Spirometry was performed as per international guidelines when the patient was able to produce acceptable and reproducible results (Beydon et al., 2007). The outcomes used in this study were the forced expiratory volume in one second (FEV_1_), forced vital capacity (FVC) and the forced expiratory flow 25%–75% (FEF_25-75_). Lung function indices were expressed as %-predicted (FEV_1_% pred, FVC % pred, FEF_25-75_% pred) calculated using the Global Lung Initiative (GLI) reference equations. The validity of these equations has been confirmed in the Australian and European populations (Quanjer et al., 2012).

### 2.7 Statistical analysis

To determine whether the clinical characteristics of exposed and unexposed subjects were significantly different at the baseline visit we used Mann-Whitney U tests. To assess the effect of CFTR modulator therapy on lung disease progression, we compared follow-up clinical outcomes of exposed and unexposed subjects based on an analysis of covariance (ANCOVA) framework implemented *via* linear regression. Models were adjusted for baseline age, research site, and the baseline value of the outcome measure being modelled (respectively referred to as baseline disease severity). No adjustments for multiple comparisons were applied; we report the coefficients from the regression models (with 95% confidence intervals) and assess significance based on an alpha of 0.05. To investigate the effect of CFTR modulator treatment in early lung disease, subgroup analyses were performed on data of children and adolescents aged <18 years. A logarithmic transformation was applied to variables that did not follow a normal distribution, as appropriate. All analyses were performed using Stata version 15.1 (Stata Corp., College Station, TX).

## 3 Results

### 3.1 Study population

In total, 41 PwCF from Leuven (12 adults, 8 children), Perth (19 children) and Rotterdam (2 children) were included in this retrospective study. We included 16 exposed subjects and 25 unexposed. Median (range) age for the paediatric subjects at the baseline visit was 9.0 (4.3–15.3) years and 8.1 (3.5–13.7) years, respectively; and for the adult subjects was 33.0 (24.7–36.5) years and 34.1 (26.0–38.3) years, respectively. Cohort demographics and clinical characteristics at baseline and follow-up visits are provided in Table 1. Two adult unexposed subjects had severe respiratory dysfunction (FEV_1_% pred <40%) at the baseline visit. Baseline clinical characteristics did not differ significantly between groups apart from PRAGMA-CF %Dis extent (*p* = 0.014), which was higher in the exposed group. Mean (range) CFTR modulator treatment duration was 0.93 (0.04–2.82) years at the time of the follow-up CT scan.

**TABLE 1:** Clinical characteristics at baseline and after initiation of CFTR modulator therapy.

	Baseline visit		Follow-up visit	
	Control (unexposed)	Treatment (exposed)	Control (unexposed)	Treatment (exposed)
Subjects, n	25	16	-	-
Paediatric subjects, n (%) (age <18 years)	19 (76%)	10 (62.5%)	-	-
**Nationality**				
Australia, n	13 ^a^	6 ^b^	-	-
Belgium, n	10 ^c^	10 ^d^	-	-
Netherlands, n	2 ^e^	0	-	-
Age (years)	8.3 (3.5, 38.3)	12.6 (4.3, 36.5)	12.4 (5.2, 42.3)	14.9 (5.8, 40.8)
Male, n (%)	13 (52.0%)	10 (62.5%)	-	-
Phe508del homozygote, n (%)	14 (56.0%)	5 (31.3%)	-	-
Gating mutation, n (%)	3 (12.0%)	11 (68.8%)	-	-
Pancreatic sufficiency n (%)	0 (0%)	3 (18.8%)	-	-
Time between CT visits (years)	3.88 (1.69, 4.98)	2.69 (0.99, 6.04)	-	-
Height (cm)	126.0 (99.6, 182.0)	152.0 (101.0, 177.0)	149.0 (108.1, 183.0)	153.5 (109.0, 179.1)
BMI (kg/m^2^)	16.7 (12.4, 24.2)	17.3 (14.2, 28.1)	17.8 (13.4, 23.6)	19.1 (15.8, 29.0)
FEV_1_% predicted	95.1 (27.6, 117.6)	77.2 (48.3, 109.9)	89.7 (31.1, 112.8)	82.3 (54.5, 129.8)
FVC % predicted	102.3 (52.4, 136.8)	93.7 (63.3, 121.4)	92.7 (60.3, 136.6)	86.7 (66.7, 126.6)
FEF_25-75_% predicted	81.9 (9.3, 130.5)	66.4 (10.3, 106.5)	68.9 (9.3, 118.2)	66.5 (19.1, 121.6)
**CF-CT score**				
Airway disease (%)	7.4 (0, 30.7)	9.7 (0.8, 28.9)	12.7 (0, 41.7)	8.2 (0.6, 32.3)
Bronchiectasis (%)	4.2 (0, 31.3)	5.6 (0, 21.2)	9.7 (0, 49.7)	7.3 (0, 29.2)
Mucus plugging (%)	16.7 (0, 38.9)	18.1 (0, 38.9)	22.2 (0, 41.7)	15.3 (0, 33.3)
**PRAGMA-CF**				
Airway disease (%)	1.6 (0, 19.7)	4.7 (0.7, 14.0)	1.8 (0, 26.3)	3.9 (0.1, 15.4)
Bronchiectasis (%)	1.2 (0, 17.5)	2.2 (0, 9.1)	0.7 (0, 23.8)	0.9 (0, 9.7)
Mucus plugging (%)	0.3 (0, 4.9)	1.6 (0, 4.9)	0.5 (0, 7.8)	0.4 (0, 5.7)
**Airway-artery dimensions**				
Airway-artery pairs, n	84 (3, 199)	44 (1, 186)	61 (6, 214)	45 (8, 126)
Outer AAR	1.7 (1.3, 2.1)	1.7 (1.2, 2.2)	1.7 (1.5, 2.1)	1.8 (1.5, 2.3)
WAR	0.9 (0.7, 1.2)	0.9 (0.5, 1.1)	0.9 (0.8, 1.0)	0.9 (0.7, 1.4)
Inner intra-branch tapering	0.6 (−0.4, 1.3)	0.7 (−0.9, 2.1)	0.7 (−1.2, 1.4)	0.7 (−0.3, 1.6)
Outer intra-branch tapering	0.6 (−0.5, 3.1)	0.6 (−0.9, 1.6)	0.6 (−0.4, 1.2)	0.6 (−0.4, 1.0)
Inner inter-branch tapering	29.3 (17.0, 40.1)	28.3 (20.3, 36.0)	29.7 (9.8, 37.9)	26.2 (−18.0, 39.5)
Outer inter-branch tapering	19.6 (3.2, 33.1)	21.3 (10.6, 45.5)	18.8 (5.1, 26.6)	18.2 (−3.8, 28.2)

Data are reported as median (range) unless otherwise indicated.

^a^ Includes 13 children.

^b^ Includes 6 children.

^c^ Includes 6 adults and 4 children.

^d^ Includes 6 adults and 4 children.

^e^ Includes 2 children.

BMI, body mass index; FVC, forced vital capacity, FEV_1_, forced expiratory volume in one second, FEF_25-75_, mid-expiratory flow rate; outer AAR, outer airway-artery ratio; WAR, wall-artery ratio.

### 3.2 CT scan characteristics

A total of 82 CT scans were available for analysis (41 paired baseline and follow-up visits) and 80 CT visits had paired spirometry measurements. Baseline CT scans were performed between 2007–2016 for exposed subjects and 2008–2014 for unexposed subjects. Slice thickness ranged between 0.9 and 5 mm. CT scan characteristics are summarised in Supplementary Table S1. Findings from these models are discussed in term below.

### 3.3 CT image analysis

CT scans were assessed using the PRAGMA-CF, AA-dimensions and CF-CT methods. Unadjusted changes in lung disease outcomes between baseline and follow-up visits for the entire cohort and paediatric subjects are provided in Supplementary Tables S2, S3, respectively. Results of the ANCOVA analysis comparing follow-up lung disease outcomes in exposed and unexposed groups are shown for the entire cohort and paediatric subjects in Tables 2, 3, respectively.

**TABLE 2:** Effects of CFTR modulator treatment on lung disease progression across all subjects. For spirometry, positive coefficients indicate improvement in lung capacity. For CF-CT and PRAGMA-CF, negative coefficients indicate improvement in lung structural changes. For airway-artery, positive outer AAR and WAR coefficients, and negative tapering coefficients indicate improvement in lung structural changes.

Outcome	Model coefficient (confidence interval) ^a^	*p*-value
FEV_1_% predicted	11.15 (2.21, 20.09)	**0.016**
FVC % predicted	0.01 (−7.90, 7.92)	0.998
FEF_25-75_% predicted	10.80 (0.45, 21.15)	**0.041**
**CF-CT score**		
Airway disease (%)	−3.72 (−7.90, 0.46)	0.079
Bronchiectasis (%)	−3.43 (−8.13, 1.27)	0.147
Mucus plugging (%)	−4.88 (−11.39, 1.64)	0.137
**PRAGMA-CF**		
Airway disease (%)	−2.88 (−4.46, −1.30)	**0.001**
Bronchiectasis (%)	−2.07 (−3.13, −1.02)	**<0.001**
Mucus plugging (%)	−0.23 (−1.22, 0.77)	0.646
**Airway-artery dimensions**		
Outer AAR	0.05 (−0.08, 0.18)	0.410
WAR	0.07 (−0.01, 0.14)	0.073
Inner intra-branch tapering	0.00 (−0.44, 0.44)	0.996
Outer intra-branch tapering	−0.03 (−0.31, 0.25)	0.817
Inner inter-branch tapering (log)	−0.01 (−0.20, 0.18)	0.882
Outer inter-branch tapering (log)	0.08 (−0.14, 0.30)	0.485

^a^ Beta coefficient (95% CI) represents the adjusted difference in follow-up measure for the exposed group, related to the unexposed group; based on a linear regression model additionally adjusted for age, site and baseline disease severity. Statistically significant effects in bold.

**TABLE 3:** Effects of CFTR modulator treatment on lung disease progression in paediatric subjects. For spirometry, positive coefficients indicate improvement in lung capacity. For CF-CT and PRAGMA-CF, negative coefficients indicate improvement in lung structural changes. For airway-artery, positive outer AAR and WAR coefficients, and negative tapering coefficients indicate improvement in lung structural changes.

Outcome	Model coefficient (confidence interval) ^a^	*p*-value
FEV_1_% predicted	7.30 (−3.18, 17.78)	0.163
FVC % predicted	−0.37 (−11.48, 10.73)	0.945
FEF_25-75_% predicted	11.46 (−3.04, 25.95)	0.115
**CF-CT score**		
Airway disease (%)	−4.05 (−9.33, 1.24)	0.126
Bronchiectasis (%)	−4.00 (−8.31, 0.32)	0.068
Mucus plugging (%)	−3.76 (−12.67, 5.14)	0.389
**PRAGMA-CF**		
Airway disease (%)	−0.20 (−1.63, 1.23)	0.778
Bronchiectasis (%)	−0.88 (−1.70, −0.07)	**0.035**
Mucus plugging (%)	0.09 (−0.97, 1.15)	0.862
**Airway-artery dimensions**		
Outer AAR	0.07 (−0.09, 0.22)	0.393
WAR	0.04 (−0.03, 0.11)	0.309
Inner intra-branch tapering	0.15 (−0.40, 0.69)	0.583
Outer intra-branch tapering	0.03 (−0.31, 0.37)	0.844
Inner inter-branch tapering (log)	0.02 (−0.22, 0.25)	0.880
Outer inter-branch tapering (log)	0.09 (−0.20, 0.38)	0.512

^a^ Beta coefficient (95% CI) represents the adjusted difference in follow-up measure for the exposed group (subjects ≤18years old), related to the unexposed group; based on a linear regression model additionally adjusted for age, site and baseline disease severity. Statistically significant effects in bold.

#### 3.3.1 PRAGMA-CF

**FIGURE 1:**
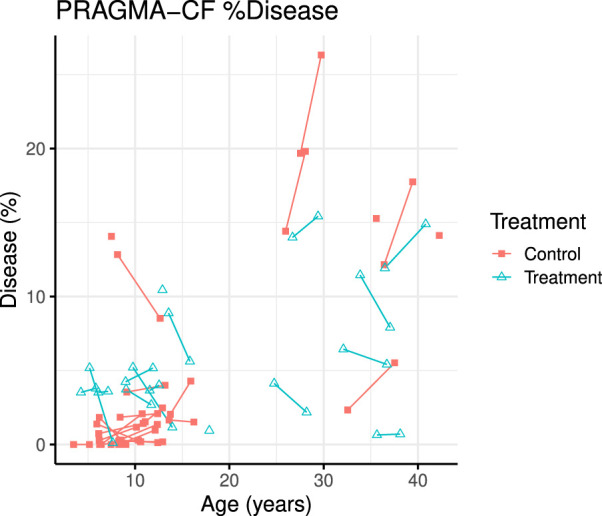
PRAGMA-CF %Airway disease over time for individual patients. Control (red squares) and treatment subjects (blue triangles) with lines joining points from the same patient.

**FIGURE 2:**
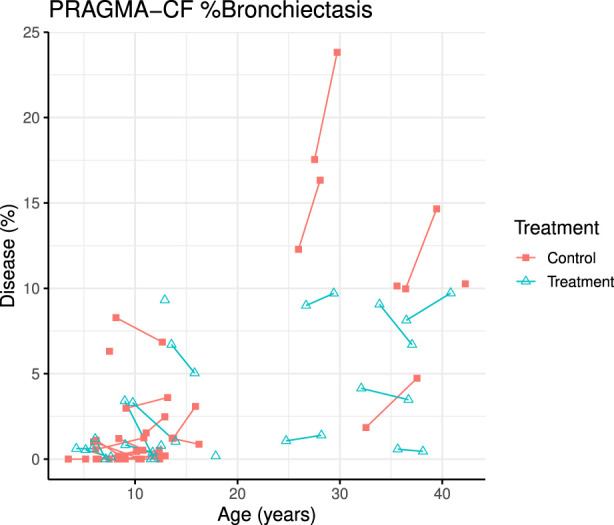
PRAGMA-CF %Bronchiectasis over time for individual patients. Control (red squares) and treatment subjects (blue triangles) with lines joining points from the same patient.

A total of 70 CT scans were analysed using PRAGMA-CF. We excluded 12 CT scans (5 baseline and 1 follow-up, and their corresponding paired scan) due to sequential or incomplete acquisition (n = 5) or substantial motion artefacts (n = 1). At follow-up, both PRAGMA-CF %Dis [-2.88 (−4.46, −1.30), *p* = 0.001] and %BE [-2.07 (−3.13, −1.02), *p* < 0.001] were lower in the exposed group compared to the unexposed (adjusted for baseline disease severity) (Table 2), indicating improvement in structural lung disease. Individual trajectories of %Dis and %BE over time are shown in Figures 1, 2, respectively. No statistically significant differences in %MP were detected between exposed and unexposed groups. In paediatric subjects (Table 3), %BE extent improved in exposed subjects [-0.88 (−1.70, −0.07), *p* = 0.035] compared to unexposed. %Dis [-0.20 (−1.63, 1.23), *p* = 0.778] and %MP [0.09 (−0.97, 1.15), *p* = 0.862] were not significantly different between exposed and unexposed subjects.

#### 3.3.2 Airway-artery dimensions

A total of 5781 AA-pairs (small airways) were computed from 75 scans. Centrelines could not be drawn for 7 scans due to sequential or incomplete acquisition of scans, extensive atelectasis, mucus plugging and/or severe bronchiectasis. Median number of AA-pairs (small airways) with AAR >1.1 at the baseline visit was 40 in exposed subjects and 70 in unexposed. There were no significant differences in outer AAR [0.05 (−0.08, 0.18), *p* = 0.410], WAR [0.07 (−0.01, 0.14), *p* = 0.073] and any tapering outcomes between exposed and unexposed subjects (Table 2). The outer AAR and WAR remained relatively stable in exposed subjects between baseline and follow-up visits (Table 1). In paediatric subjects there were no significant differences between the exposed and unexposed subjects for any AA outcomes.

#### 3.3.3 CF-CT score

In total, 72 CT scans were assessed using the CF-CT score. We excluded 10 CT scans (4 baseline and 1 follow-up, and their corresponding paired scan) due to sequential or incomplete acquisition (n = 5). Across the entire cohort, there were no significant differences in %Dis [-3.72 (−7.90, 0.46), *p* = 0.079], %BE [-3.43 (−8.13, 1.27), *p* = 0.147] or %MP [-4.88 (−11.39, 1.64), *p* = 0.137] between exposed and unexposed subjects (Table 2). Likewise, in the subgroup analysis of paediatric data, there were no statistically significant differences in %Dis, %BE or %MP extent between exposed and unexposed subjects.

### 3.4 Pulmonary function measures

In exposed subjects FEV_1_% pred [11.15 (2.21, 20.09), *p* = 0.016] and FEF_25-75_% pred [10.80 (0.45, 21.15), *p* = 0.041] improved compared to unexposed (Table 2). FVC % pred was not significantly different between exposed and unexposed groups. Subgroup analysis of paediatric data showed that there were no significant differences in pulmonary function between exposed and unexposed subjects.

## 4 Discussion

This retrospective study was performed to evaluate the ability of different quantitative CT outcomes to assess the effect of CFTR modulators on structural lung disease. We found that both CT and spirometry were able to detect treatment effects in exposed subjects relative to unexposed. Previous studies on CFTR modulators have used various clinical outcomes such as BMI (De Boeck et al., 2014; Wainwright et al., 2015), sweat chloride concentration (Boyle et al., 2014; Rosenfeld et al., 2018), pulmonary function (Ramsey et al., 2011; Ratjen et al., 2017) and pulmonary exacerbation rate (Flume et al., 2018; McColley et al., 2019) to monitor treatment efficacy. Another recent study using artificial intelligence also found treatment effects on CFTR-exposed patients (Dournes et al., 2021). Structural lung disease commences early in PwCF (Sly et al., 2009; Mott et al., 2012) and is the major determinant of morbidity and mortality (Davis, 2006). Therefore, we believe that quantitative CT assessment of lung structure is an important method to study the effect of CFTR modulator therapy on structural lung abnormalities related to CF lung disease.

CFTR modulator therapy was associated with improvements in structural lung disease and bronchiectasis assessed using PRAGMA-CF. In paediatric subjects, only PRAGMA-CF %BE extent improved following treatment. Correcting CFTR dysfunction facilitates increased hydration of the airway surface and thinning of pulmonary secretions (e.g., mucus), leading to improved mucociliary clearance. These changes may result in reduced pulmonary infection and inflammation and improvement in structural lung disease. Although studies have shown that CFTR modulators improve mucociliary clearance (Donaldson et al., 2018), we did not detect any significant differences in mucus plugging extent between exposed and unexposed groups. This could be because our cohort primarily consisted of children (median age of 8.3 years at baseline), who generally have early stages of lung disease and low levels of mucus plugging (Rosenow et al., 2015). In addition, PRAGMA-CF is performed in a hierarchical manner, with bronchiectasis taking precedence over other structural lung abnormalities. Although the extent of mucus plugging was underestimated using this method, PRAGMA-CF %Dis improved in exposed subjects compared to unexposed. This most likely reflects a reduction in airway wall thickness due to improved mucus clearance. As treatment was associated with improvements in both bronchiectasis and non-bronchiectatic lung abnormalities, this suggests that CFTR modulators may benefit patients with a range of lung disease severity. However, a longitudinal study of extended duration is needed to confirm these findings.

CFTR modulator therapy was not associated with a significant improvement in any AA outcome: outer AAR, WAR or airway tapering. As PRAGMA-CF %Dis has been reported to be the most sensitive predictor of AA-dimensions in PwCF (Kuo et al., 2017c), we expected exposed subjects to have lower outer AAR and WAR, and increased airway tapering compared to unexposed. Although PRAGMA-CF %BE improved following therapy there were no significant differences in outer AAR, WAR or tapering (inter- and intra-branch) in our cohort. This discrepancy may be due to variability in lung volume control during CT acquisition. As bronchiectasis is considered an irreversible structural change, the reduction in %BE is likely to be related to decreased visibility of widened airways in the periphery of the lung. In addition, PRAGMA-CF %Dis consists of irreversible (e.g., bronchiectasis) and reversible (e.g., airway wall thickening) structural lung abnormalities (Chang et al., 2018) that may improve or progress at different rates in response to treatment. We found that the outer AAR, WAR and PRAGMA-CF %BE remained relatively stable in exposed subjects between baseline and follow-up visits. This suggests that CFTR modulators may potentially attenuate further progression of bronchiectasis and reverse airway wall thickening and dilation in PwCF. In comparison, Sheikh et al. (2015) reported significant improvements in structural lung disease (bronchiectasis, mucus plugging and airway wall thickening) following Ivacaftor, using the semi-quantitative CF-CT score. However, their study was performed in an older cohort (mean age of 20.9 years), where lung disease is more severe and easier to detect changes using less sensitive methods.

Although CFTR modulator therapy was associated with improved FEV_1_% pred and FEF_25-75_% pred, baseline spirometry characteristics were different between exposed and unexposed groups. As study subjects were retrospectively selected, we cannot exclude the possibility of sampling bias where exposed subjects may have had worse respiratory health compared to unexposed when spirometry was performed. Therefore, statistical significance is possibly due to regression toward the mean. However, it is unlikely due to sampling bias because participating clinics follow a standardised protocol to monitor PwCF. Statistical models were also adjusted for baseline disease severity so differences in clinical characteristics between exposed and unexposed groups is unlikely to have a large effect on our results. Unlike spirometry and CF-CT, structural lung disease assessed with PRAGMA-CF improved in children. This suggests that quantitative CT biomarkers are more sensitive to treatment effects than spirometry and semi-quantitative CT methods.

This study has various limitations including the lack of a true control (placebo) group and the inclusion of children, adolescents and adults in the same cohort. The limited sample size is a further limitation. To reliably assess the effect of CFTR modulators in paediatric patients a larger number of study subjects is needed. Other studies on CFTR treatments also suffered from a limited sample size for CFTR-exposed patients (Dournes et al., 2021). In addition, the exposed group is composed of subjects who received ivacaftor or lumacaftor-ivacaftor. Although the statistical models were adjusted for baseline disease severity, Ivacaftor subjects generally have milder lung disease severity than lumacaftor-ivacaftor subjects and patient genotype may therefore have been a confounding variable. Due to sample size we were unable to assess the individual effects of these treatments, as the subgroup analyses were underpowered to determine statistical significance.

CT scans were performed as part of routine clinical practice and not scheduled to match the start of CFTR modulator therapy. Therefore, the treatment duration and time interval between baseline and follow-up visits greatly varied per patient. In addition, expiratory CT images were not available for the majority of subjects, so we were unable to assess the effect of CFTR modulators on CT low attenuation regions, which are an important measure of small airways disease (Tepper et al., 2013). CT acquisition at different lung volume levels (voluntary breath hold and spirometry-controlled) also affects objective evaluation of bronchiectasis and bronchial wall thickening, leading to suboptimal image analysis.

For AA-dimensions, we excluded severely bronchiectatic airways without a detectable paired artery because the analysis of bronchoarteriolar dimensions uses the artery properties as a normalising factor. Although the requirement for a paired artery limits the sensitivity of the outer AAR to detect severely bronchiectatic airways, the sensitivity of tapering outcomes is not affected because they do not require a paired artery. Although the extent of bronchiectasis may have been slightly underestimated using the AA-method, this is unlikely to affect the results of patients who already have severe lung disease. Our results also indicate that diffuse airway widening and thickening are likely to be more sensitive measures of lung disease severity in PwCF than bronchiectasis. In addition, the normalisation we used to correct for differences in lung volumes of patients in our cohort can lead to larger variance in the AA measurements. This is, when correcting for the actual lung volume in each CT in cases where there is a large difference in inspiration level between baseline and follow-up, this can lead to different small airways and arteries being counted and paired. This larger variance may explain why we observed non-significant differences in outer AAR, WAR and airway tapering in our cohort, between baseline and follow-up. Moreover, we observed a difference in the number of extracted small airways and AA-pairs between exposed and unexposed groups at baseline (small airways: 40 (17–72) compared to 74 (48–127), *p* = 0.09; AA-pairs: 134 (52–146) compared to 145 (107–192), *p* = 0.17). This difference is not significant, but it could influence the comparison of measurements between the two groups.

## 5 Conclusion

Our preliminary study suggests that quantitative CT outcomes may provide more sensitive assessments of treatment efficacy compared to semi-quantitative CT outcomes and spirometry. In children, among the evaluated methods, only PRAGMA-CF demonstrated an effect of treatment. Since CFTR modulator therapy is likely to benefit PwCF with varying degrees of structural lung disease, quantitative CT outcomes may be useful to monitor responses in the majority of individuals. A longitudinal study of extended duration in a large cohort of paediatric CF patients, and with standardized times of CT scanning, is needed to determine the long-term effects of different classes of CFTR modulators on early structural lung disease.

## Data availability statement

The original contributions presented in the study are included in the article/Supplementary Material, further inquiries can be directed to the corresponding author.

## Supplementary material

SUPPLEMENTARY FIGURE S1 Stacked histogram of PRAGMA-CF scores for all patients at baseline (*t* = 1), sorted by %Airway disease score. The stacked bars indicate, in this order, %Bronchiectasis, %Mucus plugging, %Wall thickening, and %Healthy. The bar positions for each patient match those in Supplementary Figure S2 for follow-up (*t* = 2).

SUPPLEMENTARY FIGURE S2 Stacked histogram of PRAGMA-CF scores for all patients at follow-up (*t* = 2), sorted by %Airway disease score. The stacked bars indicate, in this order, %Bronchiectasis, %Mucus plugging, %Wall thickening, and %Healthy. The bar positions for each patient match those in Supplementary Figure S1 for baseline (*t* = 1).
