# Supplementary material for: The effect of CFTR modulators on structural lung disease in cystic fibrosis
Source: Front Pharmacol. 2023 Apr 11;14:1147348. doi: 10.3389/fphar.2023.1147348 (PMC10127680; doi:10.3389/fphar.2023.1147348)
Supplement: Supplementary file 1 [file Table1.DOCX]

**Online supplement 1:** CT scan characteristics.

| **CT scan details** | **n (%)** |
| --- | --- |
| Volumetric  Sequential | 80 (97.6)  2 (2.4) |
| Pressure-controlled | 10 (12.2) |
| Spirometry-controlled | 15 (18.3) |
| Voluntary breath hold | 55 (67.1) |
| Free-breathing | 2 (2.4) |
