# Supplementary figures and images for: The effect of CFTR modulators on structural lung disease in cystic fibrosis
Source: Front Pharmacol. 2023 Apr 11;14:1147348. doi: 10.3389/fphar.2023.1147348 (PMC10127680; doi:10.3389/fphar.2023.1147348)

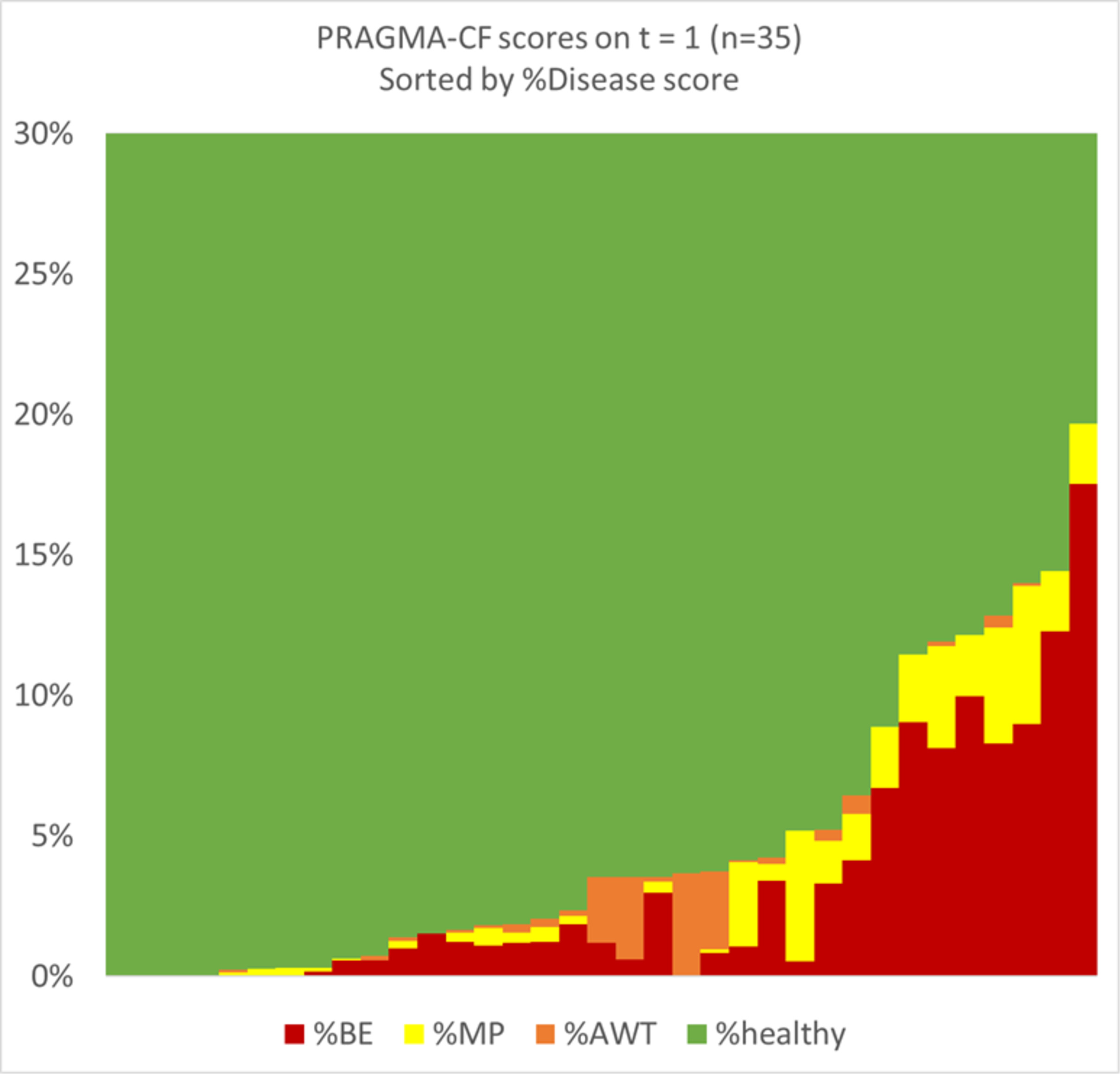

Supplement: Supplementary file 2 [file Image1.jpeg]

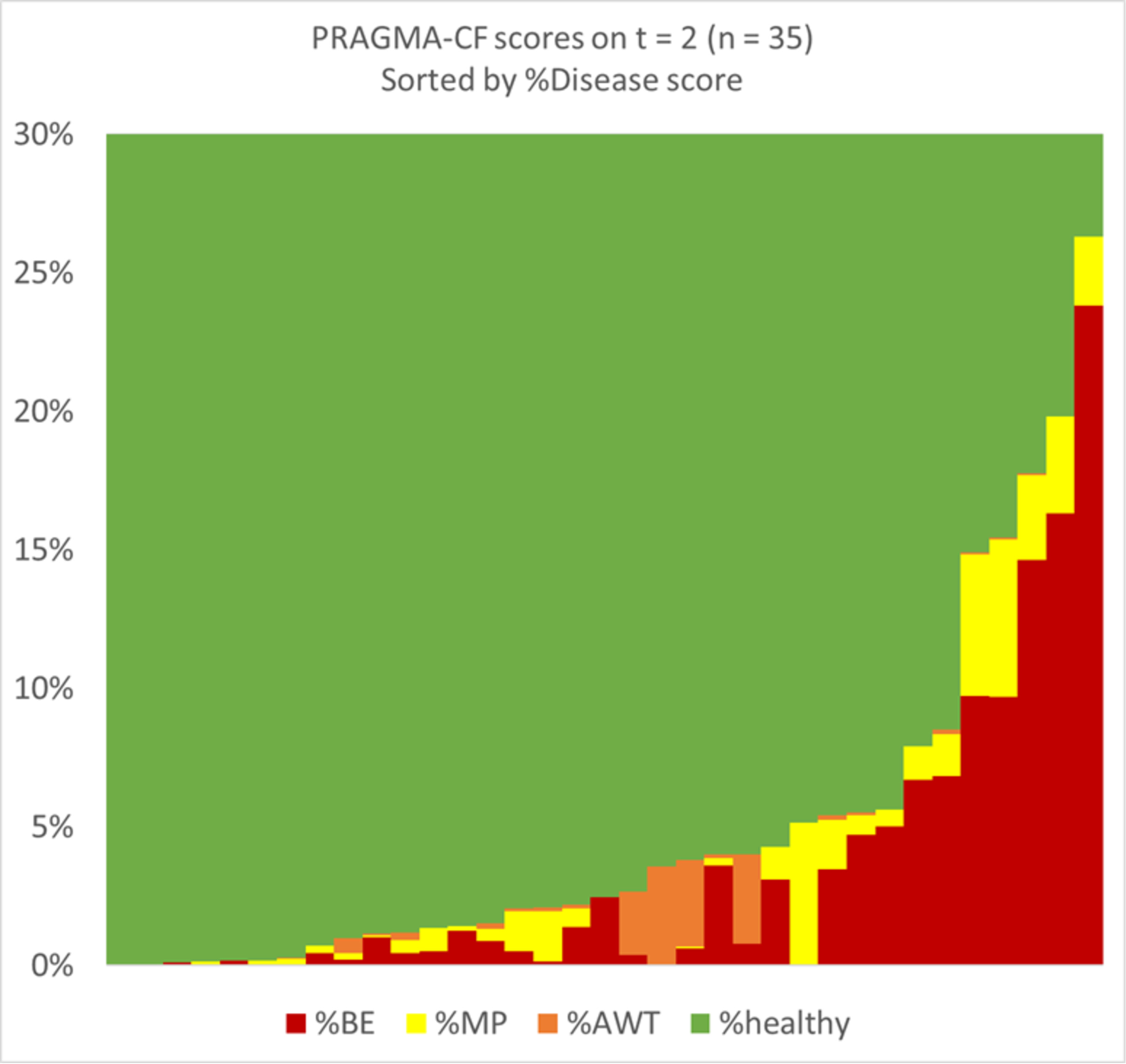

Supplement: Supplementary file 3 [file Image2.jpeg]
