# Supplementary material for: The effect of CFTR modulators on structural lung disease in cystic fibrosis
Source: Front Pharmacol. 2023 Apr 11;14:1147348. doi: 10.3389/fphar.2023.1147348 (PMC10127680; doi:10.3389/fphar.2023.1147348)
Supplement: Supplementary file 4 [file Table2.docx]

**Online supplement 2:** Clinical outcomes of all subjects after initiation of CFTR modulator treatment.

| **Outcome** | **Change from baseline (mean ± SD)** | |
| --- | --- | --- |
|  | **Control (unexposed)(n=25)** | **Treatment (exposed)(n=16)** |
| BMI (kg/m^2^) | 1.0 ± 1.6 | 1.0 ± 1.5 |
| FEV_1_ % predicted | -6.3 ± 11.8 | 5.4 ± 16.6 |
| FVC % predicted | -2.0 ± 14.0 | -1.8 ± 9.5 |
| FEF_25-75_ % predicted | -7.7 ± 16.5 | 1.5 ± 15.6 |
| **CF-CT score** |  |  |
| Airway disease (%) | 4.0 ± 6.1 | 1.6 ± 4.4 |
| Bronchiectasis (%) | 4.2 ± 7.2 | 2.9 ± 5.6 |
| Mucus plugging (%) | 2.8 ± 11.5 | -1.0 ± 9.5 |
| **PRAGMA-CF** |  |  |
| Airway disease (%) | 1.1 ± 2.5 | -1.0 ± 2.3 |
| Bronchiectasis (%) | 0.9 ± 2.0 | -0.7 ± 1.4 |
| Mucus plugging (%) | 0.2 ± 0.9 | -0.3 ± 2.1 |
| **Airway-artery dimensions** |  |  |
| Outer AAR | 0.1 ± 0.4 | 0.0 ± 0.1 |
| WAR | 0.0 ± 0.1 | 0.1 ± 0.1 |
| Inner intra-branch tapering | 0.0 ± 0.6 | 0.0 ± 0.6 |
| Outer intra-branch tapering | 0.2 ± 0.4 | 0.0 ± 0.3 |
| Inner inter-branch tapering | -0.8 ± 6.7 | 1.8 ± 7.0 |
| Outer inter-branch tapering | -0.9 ± 7.1 | 3.2 ± 6.0 |

SD: Standard deviation
