# Supplementary material for: The effect of CFTR modulators on structural lung disease in cystic fibrosis
Source: Front Pharmacol. 2023 Apr 11;14:1147348. doi: 10.3389/fphar.2023.1147348 (PMC10127680; doi:10.3389/fphar.2023.1147348)
Supplement: Supplementary file 5 [file Table3.docx]

**Online supplement 3:** Clinical outcomes of paediatric subjects after initiation of CFTR modulator treatment.

| **Outcome** | **Change from baseline (mean ± SD)** | |
| --- | --- | --- |
|  | **Control (unexposed)(n=19)** | **Treatment (exposed)(n=10)** |
| BMI (kg/m^2^) | 1.4 ± 1.6 | 1.8 ± 1.4 |
| FEV_1_ % predicted | -6.6 ± 10.7 | 6.5 ± 14.3 |
| FVC % predicted | -2.2 ± 14.2 | -0.7 ± 11.2 |
| FEF_25-75_ % predicted | -10.3 ± 16.7 | 2.5 ± 17.9 |
| **CF-CT score** |  |  |
| Airway disease (%) | 3.8 ± 6.5 | -0.3 ± 3.6 |
| Bronchiectasis (%) | 3.1 ± 5.1 | -0.5 ± 3.4 |
| Mucus plugging (%) | 3.5 ± 12.6 | -1.7 ± 10.5 |
| **PRAGMA-CF** |  |  |
| Airway disease (%) | 0.1 ± 1.5 | -1.5 ± 2.3 |
| Bronchiectasis (%) | 0.1 ± 0.8 | -1.1 ± 1.3 |
| Mucus plugging (%) | 0.1 ± 0.9 | -0.4 ± 2.6 |
| **Airway-artery dimensions** |  |  |
| Outer AAR | 0.0 ± 0.2 | -0.1 ± 0.1 |
| WAR | -0.1 ± 0.1 | 0.0 ± 0.1 |
| Inner intra-branch tapering | 0.0 ± 0.6 | 0.1 ± 0.8 |
| Outer intra-branch tapering | 0.2 ± 0.3 | 0.0 ± 0.3 |
| Inner inter-branch tapering | -1.1 ± 7.2 | 3.7 ± 8.3 |
| Outer inter-branch tapering | -1.4 ± 7.3 | 3.8 ± 7.9 |

SD: Standard deviation
